# The rubber hand illusion is a fallible method to study ownership of prosthetic limbs

**DOI:** 10.1038/s41598-021-83789-7

**Published:** 2021-02-24

**Authors:** Jan Zbinden, Max Ortiz-Catalan

**Affiliations:** 1 Center for Bionics and Pain Research, Mölndal, Sweden; 2 Department of Electrical Engineering, Chalmers University of Technology, Gothenburg, Sweden; 3 Operational Area 3, Sahlgrenska University Hospital, Mölndal, Sweden; 4 Department of Orthopaedics, Institute of Clinical Sciences, Sahlgrenska Academy, University of Gothenburg, Gothenburg, Sweden

**Keywords:** Neuroscience, Sensory processing, Outcomes research

## Abstract

Enabling sensory feedback in limb prostheses can reverse a damaged body image caused by amputation. The rubber hand illusion (RHI) is a popular paradigm to study ownership of artificial limbs and potentially useful to assess sensory feedback strategies. We investigated the RHI as means to induce ownership of a prosthetic hand by providing congruent visual and tactile stimuli. We elicited tactile sensations via electric stimulation of severed afferent nerve fibres in four participants with transhumeral amputation. Contrary to our expectations, they failed to experience the RHI. The sensations we elicited via nerve stimulation resemble *tapping* as opposed to *stroking,* as in the original RHI. We therefore investigated the effect of tapping versus stroking in 30 able-bodied subjects. We found that either tactile modality equally induced ownership in two-thirds of the subjects. Failure to induce the RHI in the intact hand of our participants with amputation later confirmed that they form part of the RHI-immune population. Conversely, these participants use neuromusculoskeletal prostheses with neural sensory feedback in their daily lives and reported said prostheses as part of their body. Our findings suggest that people immune to the RHI can nevertheless experience ownership over prosthetic limbs when used in daily life and accentuates a significant limitation of the RHI paradigm.

## Introduction

In addition to loss of function, limb amputations pose a significant threat to a person’s body image. The body image represents the perceptual, conceptual, and emotional aspects of our bodies in our mind^1^. Limb loss immediately affects the perceptual and conceptual representation; the stored structural description of the body substantially mismatches the received visual and somatosensory feedback. Moreover, the exclusion from social rituals like handshaking, and prejudicial attitudes towards disabilities, can damage the emotional aspects of the body image and lead to a negative relation towards a missing limb. A distorted body image has also been correlated with “decreased life satisfaction, quality of life, activity levels, and overall psychological adjustment”^2^. One possibility to restore a distorted body image, and its related social and psychological consequences after limb amputation, might be the use of a prosthetic limb as substitute for the lost limb.

The use of highly sophisticated prosthetic limbs, offering natural aesthetics and increased functionality to reenable participation in social life, may improve the conceptual and emotional elements of the body image. One of the most promising advances to address the third element of the distorted body image—the perceptual aspects—is the restoration of sensory feedback. Different deployed strategies to this end include sensory substitution^3,4^, targeted sensory reinnervation^5^, and direct nerve stimulation^6–8^. A prosthesis offering intuitive and natural sensory feedback could achieve the shift from being perceived as just a technical device, to replacing the missing afferent sensory information and thus restore the user’s body image.

Experimental paradigms to evaluate differences between sensory feedback strategies are needed to evaluate the efficacy of such strategies. However, directly measuring the body image—an abstracted model of how the body is presented in the mind—has proven to be difficult. Nevertheless, one route to assess a change in the body image is by studying the sense of ownership (hereinafter referred as ownership)^9^.

Ownership is an aspect of self-awareness related to experiencing parts of our body belonging to ourselves. Ownership over a body part can be experienced regardless of whether said body part is at rest or in motion, and whether or not movement is under one's owns volition (as opposed to agency). One of the most prominent theories of how ownership emerges is a neurocognitive model based on a top-down account^10^. In the example of a prosthesis, the model first compares the visual congruency of the prosthesis to a concept of a biological limb (or a prosthesis^11^) stored in the body image. In a second step, the postural features of the prosthesis are compared to the current body posture. Multisensory integration of available afferent information is the last step within the model. If all three comparators match, ownership over the prosthesis arises. This neurocognitive model was later extended with ideas of predictive coding^12^, which stipulates that if there are inconsistencies between the received sensory feedback and the predictions based on body image, for example, the representation of the body in the brain is updated to minimize said inconsistencies.

Whereas functional advantages of restoring sensory perception should be evaluated in experimental conditions as close to the real-life use of the prosthesis as possible, disentangling confounding factors could be advantageous when studying properties specifically related to sensory feedback. A non-agentic experiment excludes confounding variables such as algorithms for decoding motor volition, the fixation of the prosthesis to the residual limb, and the proficiency in using a prosthetic device. These variables would otherwise play a considerable role in how a prosthesis is perceived and thereby influence prosthetic ownership. An established method to examine ownership in a non-agentic setting is the rubber hand illusion (RHI)^13^, where two brushes are used to stimulate both a rubber- and a participant’s own hand with brush strokes. The biological hand is shielded from view during the experiment, so that the rubber hand visually appears to replace the biological hand. If the visuo-tactile stimuli were perceived synchronously (this happens when the two brushes stroke synchronously) most participants reported ownership over the rubber hand. Asynchronous stimulation, however, does not lead to ownership reports of the rubber hand.

An increasing number of studies have shown that the ownership sensation can be evoked in amputees using an adapted version of the RHI experiment. Stimulation using a brush^14^ and vibrators^15^ on the residual limb, as well as mechanical and vibratory feedback on reinnervated tissue^16^, has been reported to allow for subjects with amputations to experience ownership of a rubber hand. Studies on using brain stimulation^17^, light beams^18^, and temperature^19^ demonstrated that the same ownership effects can be achieved by artificially evoked tactile feedback. Recently, two case studies with a total of three subjects with a transradial limb amputation reported increased ownership due to direct nerve stimulation during an adapted RHI experiment^20,21^. Independent replication of these findings is still necessary to establish the prevalence of such phenomenon, particularly considering the low number of subjects studied. In addition, whether similar findings can be observed in higher amputation levels remains an open question.

In this study, we explored the viability of the RHI paradigm as a tool to measure prosthetic ownership in subjects with transhumeral amputation. We recruited four participants who had been implanted with extra-neural electrodes on their severed nerves as part of a novel neuromusculoskeletal prosthetic system^8,22^. Stimulation of their afferent nerves produced discernable and localized tactile sensations on their phantom hand occupying the space of their prosthetic one^8,22–25^. However, elicited sensations via nerve stimulation are not experienced as nuanced or natural as the stroking of a brush^24^, which is the tactile stimulation normally used to induce the RHI. The perceptual experience elicited by direct nerve stimulation was reported by our participants as a gentle touch or tap on the phantom hand. We attempted to induce the RHI by providing direct nerve stimulation, while the prosthesis was tapped in the perceived location by a robotic tapping device. Contrary to the participants’ reports of strong ownership of their prosthesis in their daily life^26^, we found that the adapted RHI experiment did not result in induced ownership. We then investigated if tapping as opposed to stroking the rubber hand in 30 able-bodied subjects resulted in induced ownership. Our results indicated that the RHI could be induced comparably well with either stimulus. However, 33% of the subjects did not experienced the RHI with either tactile stimulation modality. The possibility remained that our prosthetic participants belong to the non-respondent group, independently of their amputation. We then confirmed this was the case since the RHI could not be induced in their intact hand. Our investigations suggest that the RHI is not a universally reliable paradigm to study prosthetic ownership, and thus more inclusive experimental paradigms are needed to evaluate and improve sensory feedback strategies.

## Methods

### Experiment 1: RHI in subjects with amputation

#### Subjects

Four participants with transhumeral amputation who received an implanted neuromusculoskeletal interface^8,22^ were recruited for this experiment. The individual participant is referred to by their internal patient IDs, e.g. AR007, hereafter (see Table 1). All participants use an artificial limb controller (ALC)^27^ integrated into a commercial prosthetic arm for control and sensory feedback in daily life. Neurostimulation built into the ALC allows for current-controlled stimulation pulses (see Tables 2 and 3) to be sent to the implanted cuff electrode to elicit somatosensory feedback. Informed consent in accordance with the Declaration of Helsinki as well as informed consent to publish identifying information was obtained before conducting the experiments from each subject. The study was approved by the Regional Ethical Review Board in Gothenburg (#769–12) and carried out in accordance with the declaration of Helsinki.

**Table 1:** Demographic overview of the participants with amputation.

Patient ID	AR007	AR006	AL001	AL004
Sex	Male	Male	Male	Male
Year of Birth	1973	1976	1974	1968
Age at experiment	46	44	44	51
Amputation cause	Fibromatosis	Traumatic	Traumatic	Traumatic
Amputation level	Transhumeral	Transhumeral	Transhumeral	Transhumeral
Amputation side	Right	Right	Left	Left
Date amputation	2003	1997	2011	2015
Date of electrode implantation	2013–01	2017–01	2017–01	2018–12

**Table 2:** Standard stimulation parameters.

Label	Parameter	Value
A	Electrode surface area	0.0016 cm^2^
D	Duration of stimulation phase of a stimulus pulse	200 µs
D’	Duration of reversal phase of a stimulus pulse	10*D
F	Stimulation pulse frequency	30 Hz
I	Current of stimulation phase	AR007: 80 µA
		AR006: 300 µA
		AL001: 450 µA
		AL004: 500 µA
I’	Current of reversal phase	1/10*I
N	Number of pulses per train	1800
R	Train rate	0.5 Hz
w	Inter-phase delay	50 µs
P	Total application time	360 s
S	Effective stimulation time	180 s
Y	Implantation time	AR007: 5 years
		AR006: 2 years
		AL001: 2 years
		AL004: 1 year

Parameters that were individual for each participant are marked with patient IDs.

**Table 3:** Derived stimulation parameters.

Label	Derivation	Value
C	I/I′	1/10
O	S/P	1/2
Q	I*D	AR007: 16 nC
		AR006: 60 nC
		AL001: 90 nC
		AL004: 100 nC
QD	(I*D)/A	AR007: 10,000 nC/cm^2^
		AR006: 37,500 nC/cm^2^
		AL001: 56,250 nC/cm^2^
		AL004: 62,500 nC/cm^2^
T	N/F	60 s
wF	F*O	15 Hz

The different parameter describe ﻿the amplitude of stimulating and reversal phase ratio (C), the percentage of the effective stimulation time (O), the charge of the stimulating phase (Q), the charge of the stimulating phase per unit area (QD), the train duration (T), and the weighted frequency of effective stimulation (wF).

#### Experimental design

**Figure 1:**
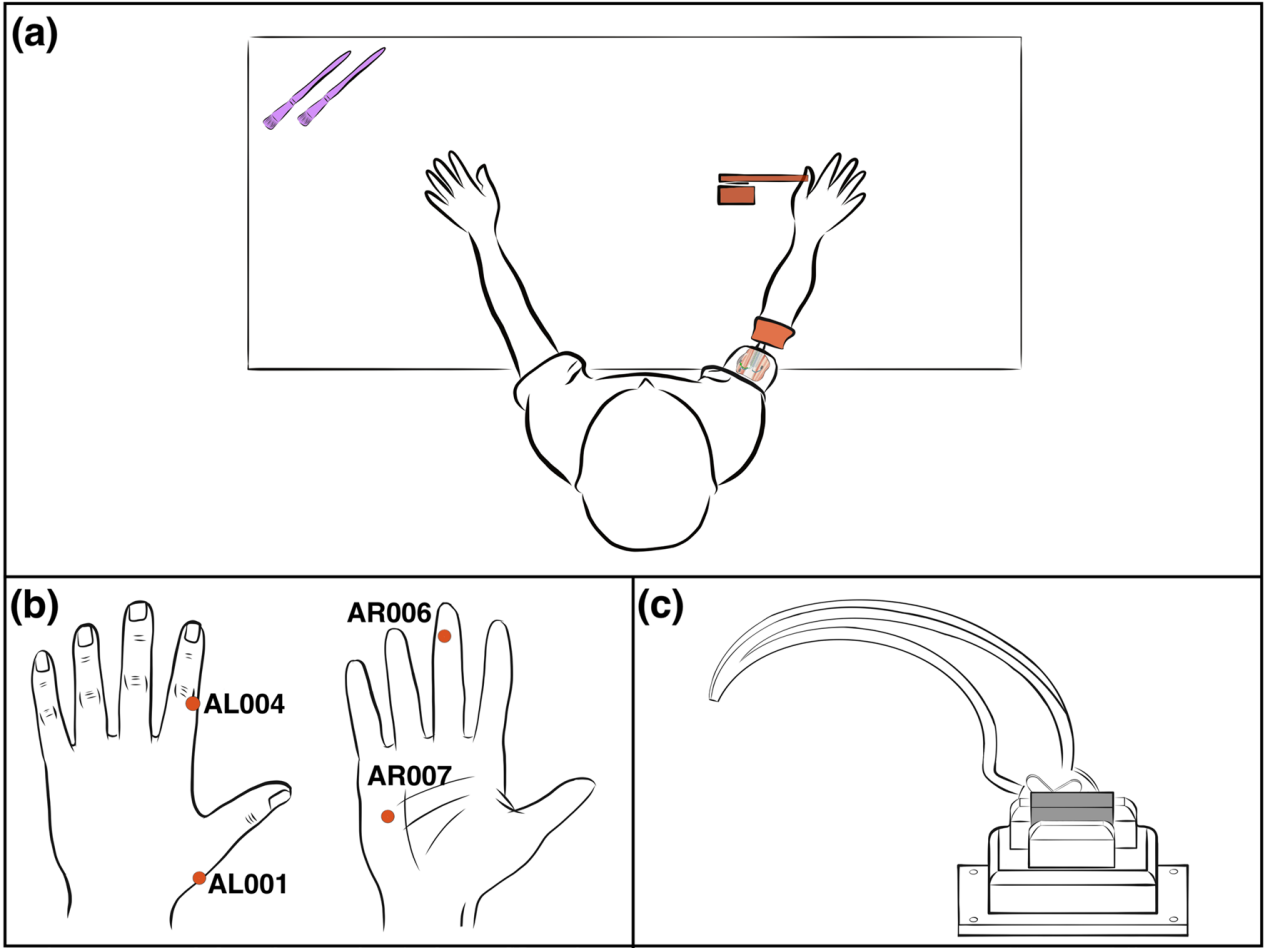
Experimental setup for participants with limb loss at the transhumeral level. (**a**) The prosthesis including the stimulation unit was connected directly to the implanted neuromusculoskeletal interface to provide neural sensory stimulation. A 3D printed tapper touched the participants’ prosthesis on the locations where the participants reported the origin of the perceived neural stimulation. (**b**) Reported perception locations in the phantom hand now occupied by the prosthetic hand (**c**) 3D printed tapper, actuated by a servomotor (grey).

Participants received neural stimulation together with visual feedback synchronously, and asynchronously as a control condition (see Fig. 1a). Elicited sensations using direct neural stimulation differ greatly from the sensation of a brushstroke as in the original RHI experiment^13^. We elicited perceptive fields on the phantom hand reported as small, confined, and relatively rounded^23^ (see Fig. 1b), whereas a brush stroke generally stimulates a larger, less defined area. Therefore, we designed a 3D printed tapping device to match the participant's visual experience to the perceptive area, quality, and duration of the sensation elicited via neural stimulation (see Fig. 1c).

Participants filled out an adapted version of the RHI questionnaire (see Table 4) after each experimental condition. The questionnaires included a Swedish translation of the questions below the original formulation. A week after the experiment, we asked the participants their agreement on questions regarding ownership at that moment (Q1, Q3, and Q4, Table 4). As an additional control condition, we conducted the conventional RHI experiment^13^ using a brush for both visuo- and tactile stimulation on the contralateral hand 2–3 weeks after the first RHI experiment using neurostimulation.

**Table 4:** The standard RHI questionnaire adapted to fit an experiment with tapping stimulation on a rubber hand.

During the experiment …	
Q1	… I felt as if the prosthetic hand was my hand
Q2	… it seemed as if the sensation I felt was caused by the tapper tapping the prosthetic hand
Q3	… it seemed like I was looking directly at my own hand, rather than at a prosthetic hand
Q4	… it seemed like the prosthetic hand was part of my body
Q5	… it felt as if my right hand was drifting towards the prosthetic hand
Q6	… it seemed as if the sensation I felt came from somewhere between my residual limb and the prosthetic hand
Q7	… it appeared as if the prosthetic hand was drifting towards my right hand
Q8	… the prosthetic hand started to change shape, color, and appearance so that it (visually) started to resemble my right hand

The first four questions are adapted from Longo et al.^64^ and are used to establish if the ownership illusion was successful. The other four questions are control statements.

#### Experiment procedure

The participant was asked to sit at the table and rest both the prosthetic as well as the contralateral arm on the table.

The neurostimulation pulse train was adjusted to stimulate for 1 s (30 pulses at a pulse frequency of 30 Hz) at 20% over the perception threshold (constant amplitude between 80 and 500 μA depending on the participant and a pulse width of 200 μs, determined similarly to Ackerley et al.^23^). The tapper was programmed to touch the prosthesis for an equal amount of time (1 s). To spatially match the elicited tactile sensation to be visually congruent to the tapper, the participants indicated the perception area resulting from the neurostimulation pulses. The tapper was then positioned to touch on the indicated area. We also calibrated the time delay between the tap and neurostimulation trigger for each participant to ensure perceived simultaneity of the feedback. The perceived simultaneity was calibrated by sending stimulation pulses ± [0, 40, 80, 100, 200, 400, 500, 600, 800, 1000] ms compared to the tap trigger command, and asking the participant to judge if the sensation was perceived before, at the time, or after seeing the tapper touching the prosthesis and thereby determining the temporal binding window^28^.

Both tactile and visual stimuli were provided once every 2 s, lasting 1 s each. During the one second without stimulation, the tapper moved up and down. In the asynchronous case, the neurostimulation was delayed by 500 ms. A complete stimulation sequence lasted 180 s. A systematic documentation of the stimulation parameters^29^ can be found in Tables 2 and 3.

The original RHI experiment on the contralateral hand was conducted for 180 s. Both conditions, synchronous and asynchronous, were tested in a random order. The following assignment was obtained for the visuo-neural condition: synchronous (AR007 and AR006) and asynchronous (AL001 and AL004) stimulation first, respectively. For the brush condition, synchronous stimulation first (AR007 and AL004) and asynchronous stimulation first (AR006 and AL001) was obtained. Noise-canceling headphones were used to mute the servo noise during the neurostimulation experiments.

### Experiment 2: RHI in able-bodied participants

#### Participants and experiment design

**Figure 2:**
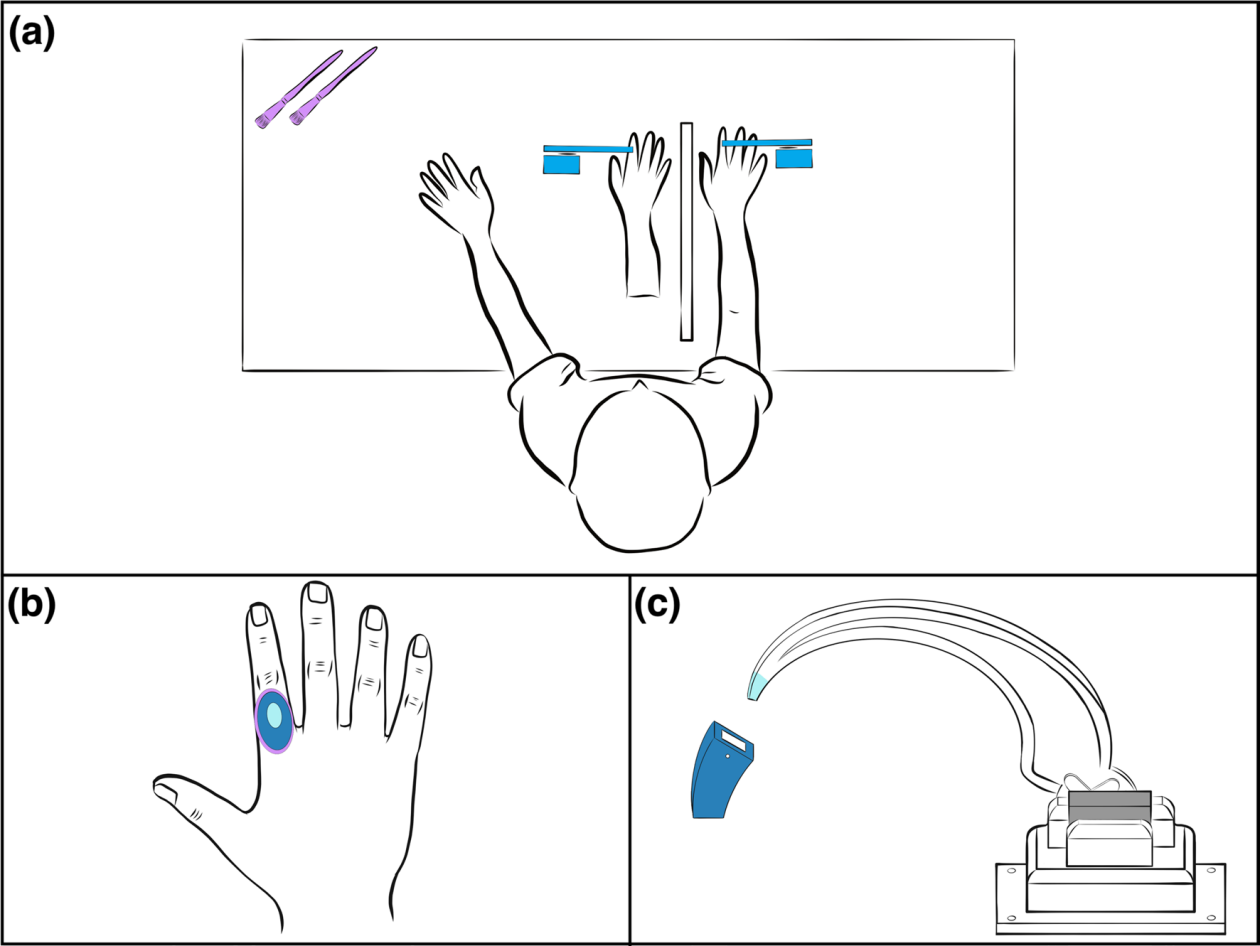
Experimental setup for able-bodied participants. (**a**) Two tappers were positioned to touch the same spots on the participant's biological hand and the rubber hand. The same setup was used for the original RHI experiment, where the tap stimulation was replaced by “stroking with a brush” stimulation. (**b**) Two different tapping stimulation areas were investigated: a small area of 1 cm^2^ (light blue area) and a larger area of 8 cm^2^ (dark blue area). For comparison: the area of a brushstroke (purple area). (**c**) The tip of the tapper was interchangeable to allow for the two different stimulation areas.

Thirty able-bodied participants were recruited on a convenience sample basis (18 male/12 female, mean age 25.0 and range 21–31). The experiment was designed to validate the use of a tapper instead of a brush within the RHI paradigm (see Fig. 2a). Additionally, we investigate the influence of the stimulation area on the sense of ownership.

#### Experiment procedure

The participant was asked to sit at the table with a rubber hand and a visual barrier already in place. They positioned their right hand next to the visual barrier in the same position as the rubber hand on the other side of the barrier. Thereafter, a blanket was used to cover both the rubber hand and the real hand from the participant’s shoulder onwards.

Two different stimulation areas were considered: a small area of 1 cm^2^ corresponding to the size of an amputee’s perceptive projected area due to neurostimulation, and a larger area of 8 cm^2^ representing the dorsal area of the proximal phalanx of the index finger, equivalent to the stimulation area of a brushstroke (see Fig. 2b). The stimulation area was changed by attaching a different tip to the 3D printed tapping device (see Fig. 2c).

The same stimulation timing as in the experiments with the participant with amputation was used: the participant received visuo-tactile stimuli once every 2 s, lasting 1 s each, for a total of 180 s. In the asynchronous condition, one tapper was delayed by 500 ms. For the asynchronous tap condition, only the small area was used to reduce the time investment for the participants.

The brush conditions were conducted with the same timing. All conditions were tested in a random order. The participants filled in the questionnaire after every condition.

### Data analysis

An ownership score^30^ for each participant was defined from the ownership statements. The ownership score was defined as:

$$OwnershipScore = mean(Q1, Q2, Q3, Q4)$$

where Q1, Q2, Q3, and Q4 are the responses to the respective questions of the questionnaire.

Due to the ordinal nature of the data obtained from the Likert scale answers of the questionnaire, computing the average of multiple questions is only mathematically justifiable if the correlation of the questions average is an accurate estimate of the average correlation of all questions. Therefore, adequate reliability and internal consistency of the four questions were determined by calculating the Cronbach’s alpha^31^ before calculating the ownership score of the able-bodied participants.

The statistical analyses comparing the ownership scores between the synchronous and asynchronous conditions for both the group of subjects with amputation as well as for the group of able-bodied subjects was conducted using the Wilcoxon Signed Rank Test, as implemented in the “*signrank*” function of MATLAB’s Statistics toolbox ﻿(The Mathworks, Inc., Natick, MA, USA). The same analysis was employed to compare the ownership score to the mean of the control questions of the RHI questionnaire in the synchronous condition. The Bonferroni method was used to correct for the three conditions (tap on small area, tap on big area, and brush) and the significance values are reported as ${p}^{*}=p*n$, where $n=3$.

As an indication of whether an individual person perceives ownership, a criterion similar to the one used by Trojan et al.^32^ was employed: Only participants yielding an ownership score higher than 4 in the synchronous condition and at least one point less in the asynchronous condition were assumed to be susceptible to RHI. This criterion is similar to using a cut-off only based on the synchronous conditions^18,33^, with the benefit of taking suggestibility of answering questionnaires into account.

Individual differences in susceptibility to the RHI and perception of ownership had been reported^34^, especially for subjects with amputation^35^. Given our heterogeneous group of participants with amputation, we also investigated the individual differences between each participant with amputation and the portion of the able-bodied control group fulfilling the ownership criterion. Similar to Bruno et al.^36^, the individual differences were evaluated with means of the Crawford’s test^37^ using the “*ttest2*” function of MATLAB’s Statistics toolbox.

The equivalence of the tapping condition compared to the brushing condition was evaluated using the Bayesian version of the Wilcoxon Signed Rank Test as implemented in JASP^38^. The standard prior was chosen, described by a zero centered Cauchy distribution with a width parameter of 0.707.

## Results

**Figure 3:**
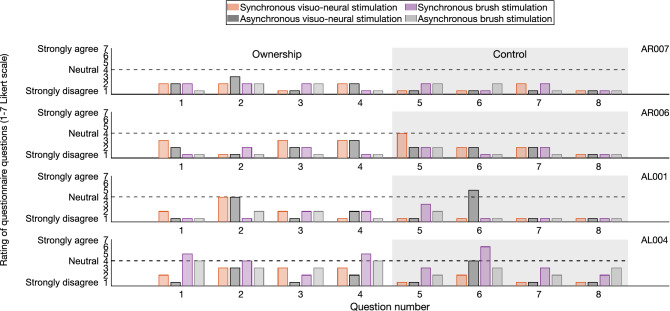
Questionnaire answers by four participants with transhumeral amputation after both the synchronous and asynchronous administration of visuo-tactile neural stimulation involving their prosthesis, and visuo-tactile feedback involving a rubber hand and their intact contralateral hand. Questions 1–4 inquired ownership over the prosthesis, whereas questions 5–8 were control questions. The dotted line indicates the mid-point of the Likert scale.

The four participants with amputation reported low ownership towards their prosthetic hand during all neurostimulation conditions (see Fig. 3), and only one assigned slightly higher questionnaire scores during the original RHI experiment in the biological limb. However, none of the participants fulfilled the ownership criterion and no significant differences were observed between the synchronous and asynchronous condition (visuo-neural: *p* = 0.375, W = 8.5; brush: *p* = 0.25, W = 6) as well as the ownership and control questions (visuo-neural: *p* = 0.125, W = 10; brush: *p* = 0.5, W = 3). These results indicate that none of the four participants is susceptible to the RHI.

**Figure 4:**
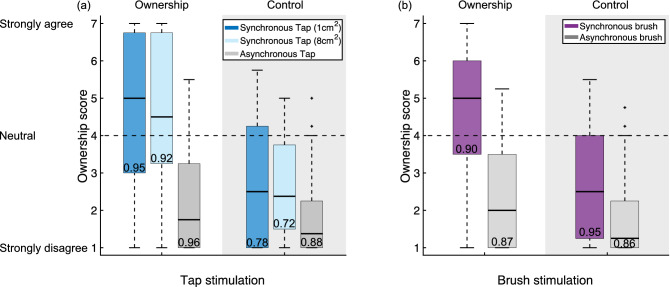
Ownership scores of the 30 able-bodied participants from (**a**) the two tap conditions and (**b**) from the brush condition. Synchronous tapping on a small area was equivalent compared to the brushing condition $$(B{F}_{10}=0.195)$$. Tapping on a bigger area is only anecdotally equivalent to brushing $$(B{F}_{10}=0.345)$$ and resulted more likely in smaller ownership scores ($$B{F}_{-0}=0.10)$$. At the bottom of each box, the Cronbach alpha is reported.

In the experiment with able-bodied participants, both tapping conditions (see Fig. 4a) show a statistically significant difference in the ownership score when comparing the respective asynchronous condition to the synchronous one (${p}^{*}$ = 1.47e−5, W = 403.5 and ${p}^{*}$ = 1.65e−5 and W = 378 for the tap on small area, and tap on big area, respectively). The ownership score is also significantly different compared to the control questions (${p}^{*}$ = 3.2e−5, W = 372 and ${p}^{*}$ = 1.56e−4 and W = 380.5 for the tap on small area, and tap on big area, respectively). Similarly, the ownership score in the brushing condition (see Fig. 4b) is significantly higher in the synchronous condition compared to the asynchronous condition (${p}^{*}$ = 8.44e−6 and W = 434) and significantly higher than the control questions (${p}^{*}$ = 6.09e−5 and W = 390). These results imply that tapping is a viable stimulation method to induce the RHI.

Out of the 30 able-bodied participants, 10 (33%), 15 (50%), and 13 (43%) did not report ownership towards the rubber hand during the tap on small area, tap on big area, and brush condition.

Comparing the individual ownership scores of the participants with amputation in the synchronous visuo-neural stimulation condition, to the group of able-bodied participants reporting ownership in the tapping condition with the same stimulation area, it was found that all four participants with amputation scored significantly lower (AR007 $p$ = 3.8e−4, AR006 $p$ = 2.1e−3, AL001 $p$ = 1.2e−3, and AL004 $p$ = 3.7e−3). Doing the same comparison on the brush conditions revealed significantly lower ownership scores for three of the participants with amputation (AR007 $p$ = 8.0e−4, AR006 $p$ = 4.7e−4, and AL001 $p$ = 4.7e−4). For AL004, the ownership score of 4 in the brushing condition is lower than the average score of 5.8 in the able-bodied control group, but not significantly lower ($p$ = 0.085). Considering the results of the ownership criterion, which takes the high ownership score of AL004 during the asynchronous control condition into account, this analysis of individual differences further supports the above results that none of the four participants is susceptible to the RHI.

**Figure 5:**
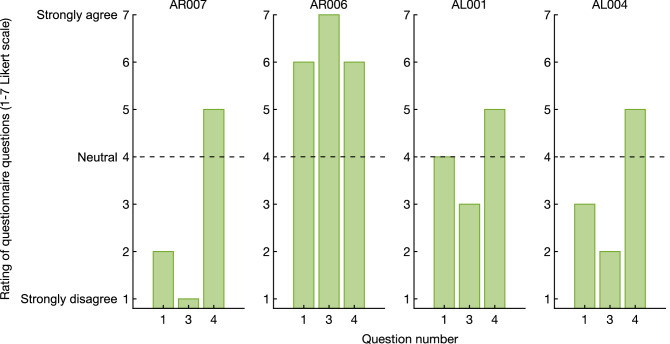
Ownership towards the prosthesis during daily life. All four participants with amputation reported their prosthetic hand as being part of their body. Q1: I feel as if the prosthetic hand is my hand, Q3: When I look at my prosthetic hand, it seems as I look directly at my own hand, rather than a prosthetic hand, Q4: The prosthetic hand is part of my body.

The questionnaire answered at home showed that all four participants with amputation experienced higher ownership over their prosthetic hand during their daily life compared to during the RHI paradigm (see Fig. 5).

## Discussion

As originally conceived, our study aimed to provide further evidence on the RHI experiment as a common paradigm to elicit ownership over a prosthetic limb. Similar to other researchers in the neuroprosthetics community, we regarded the RHI as a preferred test to evaluate the effect of sensory feedback. We expected that our participants with amputation would report ownership of their prosthesis, particularly considering recent reports of three subjects with transradial amputations for whom ownership via the RHI was induced after synchronous visuo-tactile neural stimulation^20,21^. However contrary to our expectations, none of our subjects reported ownership of their prosthetic hand during any of the experimental conditions in the RHI.

To ensure that our experimental design was not the reason for the unexpected lack of ownership, we verified the non-visuo-neural part of our setup with able-bodied participants. Our results showed that tapping is a viable stimulation method to induce ownership of a rubber hand in able-bodied subjects. This finding is consistent with contemporary work by Shehata et al*.*^39^, where tapping was used to induced ownership of a passive prosthesis worn by able-bodied participants. Furthermore, we showed that a small stimulation area was sufficient for a successful RHI. Indeed, such small area of perceived tactile stimulation was like that elicited area perceived by our participants via direct nerve stimulation.

Whereas the area and duration of visuo-tactile stimulation were matched, the quality of tapping on intact skin versus eliciting a tactile sensation via neural stimulation was not entirely equal. In an intact hand, there are different mechanoreceptors that activate differentially during touch creating a rich sensory experience that cannot be reproduced by contemporary neural electrodes, regardless of their invasiveness^24^. This difference in expected quality could have been the cause for failing to induce ownership, although other groups have reported it possible using a single stimulation contact and producing similarly large perceptive fields^20,21^, as we have done here.

Difficulties to induce the RHI has been reported on subjects with high manual dexterity, such as professional pianist^40^. This indicates that the more neural resources are dedicated to our hands, the harder is for our brain to be “fooled” by the RHI. In subjects with unilateral amputations, as our participants, control and sensory perception in the remaining hand would be potentially more developed as this is the only hand available to the subjects. This more extensive use of their able hand could potentially explain why our participants were not susceptible to the RHI in their able hand. However, by the same logic, it should be easier for them to experience the RHI in the missing hand as this is considerably less utilized. Either way, sensorimotor acuity mediating the RHI points to another potential complication on its application to subjects with amputations.

Over thirty percent of our able-bodied subjects reported no ownership towards the rubber hand in the synchronous tapping and brushing conditions. This agrees with the literature on the reported susceptibility to the RHI (22%^41^, 22%^42^, 26%^43^). Whereas subject compliance could be a reason for previously reported RHI using nerve neurostimulation^20,21^, the most likely explanation of the discrepancy with our work could be that our four subjects are among the non-negligible minority insusceptible to the RHI. Given that none of our participants with amputation reported ownership during the original RHI experiment in their contralateral intact hand, it is unlikely that the RHI would work with other stimulation means in their missing hand. These findings emphasize an impeding limitation of the RHI experiment as general paradigm to test sensory feedback strategies: the RHI paradigm can only be used with a subset of the population. Misleading conclusions on the effects of sensory feedback strategies could be drawn if one does not control for the susceptibility to the RHI in the first place.

Quantifying ownership is another hurdle in the RHI and in the study of ownership itself. We used the preferred method for quantifying ownership in the RHI (a questionnaire) to compare the experience to daily life. Yet this is far from ideal, as only three questions were relevant for such a scenario, and although the reporting scores were higher in comparison with the RHI, only one was reported consistently above the neutral value (Q4: “*The prosthetic hand is part of my body*”). Questions answerable by a numeric rating scale miss in the nuances of such a complex experience as ownership, and psychological factors might be at play on the perception of a rubber hand versus a prosthesis. Moreover, recent findings indicate that the RHI might be due to a suggestion effect^44^ and that the RHI questionnaire does not measure ownership but instead measures the ability to generate an experience to meet expectancies arising from suggestion^45^.

Three out of our four participants participated in an in-depth, qualitative investigation into the social and psychological consequences of living with neuromusculoskeletal prosthesis^26^. Said investigation found that using a highly integrated prosthesis with reliable control and sensory feedback results in a strong sense of ownership (or identification with the prosthesis as part of one’s own body)^26^. The failure to induce the RHI in subjects who otherwise report ownership of their prosthesis in their daily lives is a contradiction with farther-reaching implications. It has been assumed that demonstrating ownership in a laboratory setting justifies claims of ownership once these devices leave the experimental controlled environment. Our findings indicate that this is not a prerequisite, and how well induced ownership via RHI-like experiments in the laboratory translates to real-life usage of artificial limbs has yet to be investigated. In addition, closer examination of the sense of ownership indicates that it is dynamic, fleeting, and intensely dependent on the context of the particularly human–machine relationship in question^26^.

The discrepancy between the degree of ownership reported by our participants during daily life compared to after the RHI paradigm could be related to the deliberate non-agentic setting of the RHI experiment. The participants use their prosthesis all day and everyday^22^, and therefore routinely perform activities of daily living where they execute willed motor tasks with their prosthesis. The sense of agency that arises when the planned motor action is executed as intended has been found to strengthen the sense of ownership, when both the sense of agency and ownership co-occur^46^. The implanted neuromusculoskeletal interface used by our participants is based on osseointegration. Thus, another factor could be the additional sensory feedback received over osseoperception while moving the prosthesis^47^. Osseoperception conveys additional sensory information and can thereby complement the input received from isolated direct nerve stimulation^48,49^. Alternatively, the decrease ownership during the experiments could be attributed to the frequent don/doffing of the prosthesis in a laboratory setting. Taking of the prosthesis leads to an adjustment of the body image as the stored structural description of their body abruptly changes where, for example, the locational synchrony of the phantom limb is disrupted^26^.

Despite questionnaires being the most common assessment tool for ownership, the comparison of RHI paradigm and the daily life situation was limited in our study as no other tools were employed to assess ownership. Therefore, we cannot exclude the possibility, however unlikely, that the RHI illusion indeed induced ownerships but we could not capture its effect. Additional measures for future studies could include neural activity^50^, muscle activation^51,52^, or interoceptive sensitivity^53^. As well as more exhaustive questionnaires covering a wider range of common experiences as prosthetic user^26,54,55^ and normalization of phantom limb length^20,54,56^. On the other hand, proprioceptive drift and event-related changes in skin conductance have been found to be potentially misleading with regards to ownership^35,57–59^.

All the above considered, the RHI paradigm could still be a valuable tool to investigate sensory feedback strategies, as long as participants are susceptible to the illusion to beginning with, and its limitations are considered when assessing experimental outcomes. To strive for a more generable suite of experiments which does not exclude a subgroup of participants, we suggest including alternative paradigms that feature daily life situations (preferably in the long term), tasks investigating body-related sensorimotor integration^60^, and functional tests^61^. However, functional tests might include other aspects inducive to ownership, and therefore special care must be taken to not conflate concepts of agency with ownership^46,62^. Furthermore, the temporal nature of a sense of ownership must be considered as an important factor in examining the extent to which one can be said to embody their prosthesis^26,63^.

## Data Availability

Data can be made available by contacting the authors under reasonable requests.

## References

[1] Gallagher S (1986). Body image and body schema: A conceptual clarification. J. Mind Behav..

[2] Rybarczyk B, Behel J, Gallagher P, Desmond D, MacLachlan M (2008). Limb loss and body image. Psychoprosthetics.

[3] Cipriani C, Dalonzo M, Carrozza MC (2012). A miniature vibrotactile sensory substitution device for multifingered hand prosthetics. IEEE Trans. Biomed. Eng..

[4] Antfolk C (2013). Sensory feedback in upper limb prosthetics. Expert Rev. Med. Devices.

[5] Kuiken TA, Marasco PD, Lock BA, Harden RN, Dewald JPA (2007). Redirection of cutaneous sensation from the hand to the chest skin of human amputees with targeted reinnervation. Proc. Natl. Acad. Sci. USA..

[6] Tan DW (2014). A neural interface provides long-term stable natural touch perception. Sci. Transl. Med..

[7] Raspopovic S (2014). Restoring natural sensory feedback in real-time bidirectional hand prostheses. Sci. Transl. Med..

[8] Ortiz-Catalan M, Håkansson B, Brånemark R (2014). An osseointegrated human-machine gateway for long-term sensory feedback and motor control of artificial limbs. Sci. Transl. Med..

[9] Synofzik M, Vosgerau G, Newen A (2008). I move, therefore I am: A new theoretical framework to investigate agency and ownership. Conscious. Cogn..

[10] Tsakiris M (2010). My body in the brain: A neurocognitive model of body-ownership. Neuropsychologia.

[11] Maimon-Mor RO, Makin TR (2020). Is an artificial limb embodied as a hand? Brain decoding in prosthetic limb users. PLoS Biol..

[12] Tsakiris M (2017). The multisensory basis of the self: From body to identity to others. Q. J. Exp. Psychol..

[13] Botvinick M, Cohen J (1998). Rubber hands ‘feel’ touch that eyes see Illusions. Nature.

[14] Ehrsson HH (2008). Upper limb amputees can be induced to experience a rubber hand as their own. Brain.

[15] D’Alonzo M, Clemente F, Cipriani C (2015). Vibrotactile stimulation promotes embodiment of an Alien hand in amputees with phantom sensations. IEEE Trans. Neural Syst. Rehabil. Eng..

[16] Marasco PD, Kim K, Colgate JE, Peshkin MA, Kuiken TA (2011). Robotic touch shifts perception of embodiment to a prosthesis in targeted reinnervation amputees. Brain.

[17] Collins KL (2017). Ownership of an artificial limb induced by electrical brain stimulation. Proc. Natl. Acad. Sci..

[18] Durgin FH, Evans L, Dunphy N, Klostermann S, Simmons K (2007). Rubber hands feel the touch of light. Psychol. Sci..

[19] Kammers MPM, Rose K, Haggard P (2011). Feeling numb: Temperature, but not thermal pain, modulates feeling of body ownership. Neuropsychologia.

[20] Rognini G (2019). Multisensory bionic limb to achieve prosthesis embodiment and reduce distorted phantom limb perceptions. J. Neurol. Neurosurg. Psychiatry.

[21] Page DM (2018). Motor control and sensory feedback enhance prosthesis embodiment and reduce phantom pain after long-term hand amputation. Front. Hum. Neurosci..

[22] Ortiz-Catalan M, Mastinu E, Sassu P, Aszmann OC, Brånemark R (2020). Self-contained neuromusculoskeletal arm prostheses. N. Engl. J. Med..

[23] Ackerley R, Backlund Wasling H, Ortiz Catalan M, Brånemark R, Wessberg J (2018). Case studies in neuroscience: Sensations elicited and discrimination ability from nerve cuff stimulation in an amputee over time. J. Neurophysiol..

[24] Ortiz-Catalan M, Wessberg J, Mastinu E, Naber A, Branemark R (2019). Patterned stimulation of peripheral nerves produces natural sensations with regards to location but not quality. IEEE Trans. Med. Robot. Bionics.

[25] Ortiz-Catalan M, Mastinu E, Greenspon C, Bensmaia SJ (2020). Chronic use of a sensitized bionic hand does not remap the sense of touch. Cell Rep..

[26] Middleton A, Ortiz-Catalan M (2020). Neuromusculoskeletal limb prostheses: Personal and social implications of living with an intimately integrated bionic arm..

[27] Mastinu E, Doguet P, Botquin Y, Hakansson B, Ortiz-Catalan M (2017). Embedded system for prosthetic control using implanted neuromuscular interfaces accessed via an osseointegrated implant. IEEE Trans. Biomed. Circuits Syst..

[28] Wallace MT, Stevenson RA (2014). The construct of the multisensory temporal binding window and its dysregulation in developmental disabilities. Neuropsychologia.

[29] Günter C, Delbeke J, Ortiz-Catalan M (2019). Safety of long-term electrical peripheral nerve stimulation: Review of the state of the art. J. Neuroeng. Rehabil..

[30] Mulvey MR, Fawkner HJ, Radford HE, Johnson MI (2012). Perceptual embodiment of prosthetic limbs by transcutaneous electrical nerve stimulation. Neuromodulation.

[31] Cronbach LJ (1951). Coefficient alpha and the internal structure of tests. Psychometrika.

[32] Trojan J, Fuchs X, Speth SL, Diers M (2018). The rubber hand illusion induced by visual–thermal stimulation. Sci. Rep..

[33] Slater M (2008). Towards a digital body: The virtual arm illusion. Front. Hum. Neurosci..

[34] Riemer M, Trojan J, Beauchamp M, Fuchs X (2019). The rubber hand universe: On the impact of methodological differences in the rubber hand illusion. Neurosci. Biobehav. Rev..

[35] Niedernhuber M, Barone DG, Lenggenhager B (2018). Prostheses as extensions of the body: Progress and challenges. Neurosci. Biobehav. Rev..

[36] Bruno V, Ronga I, Fossataro C, Capozzi F, Garbarini F (2019). Suppressing movements with phantom limbs and existing limbs evokes comparable electrophysiological inhibitory responses. Cortex.

[37] Crawford JR, Garthwaite PH, Porter S (2010). Point and interval estimates of effect sizes for the case-controls design in neuropsychology: Rationale, methods, implementations, and proposed reporting standards. Cogn. Neuropsychol..

[38] JASP Team. JASP (Version 0.14) [Computer software]. (2020).

[39] Shehata AW, Rehani M, Jassat ZE, Hebert JS (2020). Mechanotactile sensory feedback improves embodiment of a prosthetic hand during active use. Front. Neurosci..

[40] Pyasik M, Salatino A, Pia L (2019). Do movements contribute to sense of body ownership? Rubber hand illusion in expert pianists. Psychol. Res..

[41] Kalckert A, Ehrsson HH (2014). The moving rubber hand illusion revisited: Comparing movements and visuotactile stimulation to induce illusory ownership. Conscious. Cogn..

[42] Ehrsson HH (2005). Touching a rubber hand: Feeling of body ownership is associated with activity in multisensory brain areas. J. Neurosci..

[43] Lloyd DM (2007). Spatial limits on referred touch to an alien limb may reflect boundaries of visuo-tactile peripersonal space surrounding the hand. Brain Cogn..

[44] Lush P (2020). Demand characteristics confound the rubber hand illusion. Collabra Psychol..

[45] Roseboom W, Lush P (2020). Serious problems with interpreting rubber hand illusion experiments. PsyArXiv.

[46] Braun N (2018). The senses of agency and ownership: A review. Front. Psychol..

[47] Clemente F (2017). Touch and hearing mediate osseoperception. Sci. Rep..

[48] Mastinu E (2019). Grip control and motor coordination with implanted and surface electrodes while grasping with an osseointegrated prosthetic hand. J. Neuroeng. Rehabil..

[49] Mastinu, E. *et al.* Motor coordination in closed-loop control of neuromusculoskeletal limb prostheses. *Sci. Rep. Under Rev.* (2020).

[50] Schmalzl L, Kalckert A, Ragnö C, Ehrsson HH (2014). Neural correlates of the rubber hand illusion in amputees: A report of two cases. Neurocase.

[51] Slater M, Perez-Marcos D, Ehrsson HH, Sanchez-Vives MV (2008). Towards a digital body: The virtual arm illusion. Front. Hum. Neurosci..

[52] Tsuji, T. *et al.* Analysis of electromyography and skin conductance response during rubber hand illusion. *Proc. IEEE Work. Adv. Robot. its Soc. Impacts, ARSO* 88–93 (2013).

[53] Tsakiris M, Tajadura-Jiménez A, Costantini M (2011). Just a heartbeat away from one’s body: Interoceptive sensitivity predicts malleability of body-representations. Proc. R. Soc. B Biol. Sci..

[54] Graczyk EL, Resnik L, Schiefer MA, Schmitt MS, Tyler DJ (2018). Home use of a neural-connected sensory prosthesis provides the functional and psychosocial experience of having a hand again. Sci. Rep..

[55] Gouzien A (2017). Reachability and the sense of embodiment in amputees using prostheses. Sci. Rep..

[56] Schmalzl L (2011). “Pulling telescoped phantoms out of the stump”: Manipulating the perceived position of phantom limbs using a full-body illusion. Front. Hum. Neurosci..

[57] Rohde M, Di Luca M, Ernst MO (2011). The rubber hand illusion: Feeling of ownership and proprioceptive drift do not go hand in hand. PLoS ONE.

[58] Abdulkarim Z, Ehrsson HH (2016). No causal link between changes in hand position sense and feeling of limb ownership in the rubber hand illusion. Attention Percept. Psychophys..

[59] D’Alonzo M, Mioli A, Formica D, Di Pino G (2020). Modulation of body representation impacts on efferent autonomic activity. J. Cogn. Neurosci..

[60] Di Pino G (2020). Sensory- and action-oriented embodiment of neurally-interfaced robotic hand prostheses. Front. Neurosci..

[61] Schiefer M, Tan D, Sidek SM, Tyler DJ (2015). Sensory feedback by peripheral nerve stimulation improves task performance in individuals with upper limb loss using a myoelectric prosthesis. J. Neural Eng..

[62] Zbinden, J., Lendaro, E. & Ortiz-Catalan, M. Prosthetic embodiment: Review and perspective on definitions, measures and experimental paradigms. *Under Rev.* 10.1186/s12984-022-01006-6 PMC8962549 35346251

[63] Murray CD (2004). An interpretative phenomenological analysis of the embodiment of artificial limbs. Disabil. Rehabil..

[64] Longo MR, Schüür F, Kammers MPM, Tsakiris M, Haggard P (2008). What is embodiment? A psychometric approach. Cognition.

